# Disseminated Tuberculosis With Granulomatous Pericarditis: The Essential Role of Forensic Histopathology in a Case of Unexpected Death

**DOI:** 10.7759/cureus.105979

**Published:** 2026-03-27

**Authors:** Ahlam Elbedwi, Hoda Tawel, Marwah Ikdeewish

**Affiliations:** 1 Forensic Histopathology, Tripoli Medical Center, Tripoli, LBY; 2 Pathology, University of Zawia, Zawia, LBY; 3 Forensic Medicine, Tripoli Medical Center, Tripoli, LBY

**Keywords:** disseminated tuberculosis, forensic histopathology, global health., granulomatous pericarditis, mycobacterium tuberculosis, postmortem diagnosis, unexpected death

## Abstract

Disseminated tuberculosis, specifically tuberculous pericarditis, represents a diagnostically challenging yet critical contributor to unexpected death in forensic practice, particularly among young migrants from endemic regions who lack access to healthcare. Forensic histopathology is often the first and only means to identify this reportable disease. Without a systematic forensic autopsy including histology, this death would have been certified as "undetermined". The present case emphasizes the necessity of improved TB screening initiatives for high-risk populations such as recent immigrants from endemic nations. We present the case of a 24-year-old male immigrant of Black ethnicity who was referred for forensic autopsy after an unexpected death. The individual was pronounced dead upon arrival at the hospital. No previous clinical history or antemortem medical data were available. External examination revealed a slim, cachectic physique with a slumped posture. Autopsy examination showed granulomatous pericarditis and extensive purulent lung abnormalities, with no signs of trauma or toxication. Histopathological analysis of the pericardium showed multinucleated giant cells, lymphocytes, epithelioid cells, and central necrosis, all of which are indicative of tuberculous pericarditis. Additionally, lung tissue examination revealed disseminated tuberculous involvement with granulomatous inflammation. The cause of death was determined to be disseminated tuberculosis, which resulted in respiratory and circulatory collapse, despite the absence of documented premortem symptoms or medical history. This case highlights the indispensable role of systematic autopsy and histopathological examination in identifying undiagnosed tuberculosis in medicolegal investigations, especially in high-risk populations. Forensic pathology enhances mortality data quality, enables contact tracing to prevent transmission, and informs targeted screening programs to strengthen TB control.

## Introduction

Tuberculosis (TB) remains a critical public health challenge worldwide, with 10.6 million new cases reported in 2022 (133 cases per 100,000 population) and 1.3 million deaths, making it the leading infectious cause of mortality worldwide [1]. While 75% of the cases are concentrated in 12 high-burden countries, such as India, Indonesia, and Nigeria, Sub-Saharan Africa follows with 45% of HIV/TB coinfections [2].

Libya, a nation with a moderate burden of TB, had an annual prevalence of 59 cases per 100,000 people (about 4,000 cases) in 2022. Nonetheless, underdiagnosis is still a problem, with only 53% of cases being identified, reflecting gaps in the diagnostic capacity of the country [1]. The escalating TB burden in Libya is caused by two interrelated factors: first, the ongoing migration from sub-Saharan African regions, and second, the significant deterioration of healthcare facilities during the COVID-19 epidemic [3,4].

Although pulmonary TB accounts for approximately 70% of the cases, disseminated TB can spread to other organs, such as the lymph nodes, pleura, kidneys, spine, brain, abdomen, liver, and heart, with various percentages [5]. Tuberculous pericarditis occurs in 1-2% of pulmonary TB cases and is discovered in roughly 1% of TB-related autopsies [6]. This active, disseminated form of disease must be distinguished from latent TB infection (LTBI), which is asymptomatic and non-infectious [7]. Unlike LTBI, Tuberculous pericarditis presents significant diagnostic and forensic challenges, particularly when it leads to sudden unexpected death in individuals without preceding cardiac symptoms [7].

*Mycobacterium (M.) tuberculosis* usually infects the pericardium either through retrograde lymphatic spread from adjacent infected lymph nodes or through hematogenous dissemination. Less frequently, the infection spreads through the blood from a distant focus or directly from adjacent lung tissue [6]. The disease advances in four stages: early inflammation with fibrin deposition, then bloody fluid accumulation, fibrous tissue creation, and lastly, scarring that stiffens the pericardium [8]. Without treatment, 30-60% of cases develop constrictive pericarditis, which causes serious consequences and increases mortality [9].

Invasive sampling is often necessary for the conclusive confirmation of the diagnosis of tuberculous pericarditis through the microbiological or histological detection of *M. tuberculosis* [8]; nevertheless, in endemic regions, clinical diagnosis often relies on supportive radiological and biochemical findings due to limited access to confirmatory testing. Granulomatous inflammation sometimes is the only observable symptom in postmortem investigations, requiring thorough histopathological analysis in addition to specialized methods like PCR and immunohistochemistry to distinguish tuberculosis from other granulomatous diseases [7]. This diagnostic approach not only establishes causation in unexpected deaths but also reveals systemic gaps in antemortem tuberculosis detection, particularly among vulnerable populations.

In the current case presentation, we highlight the crucial role of forensic pathology in identifying undetected tuberculosis through postmortem histological analysis, linking clinical medicine and public health efforts. Additionally, this instance emphasizes the need for standardized procedures for assessing granulomatous inflammation in sudden, unexpected deaths in order to enhance tuberculosis surveillance and improve mortality statistics. This case also demonstrates how forensic histopathology serves as the definitive tool for diagnosing active disseminated tuberculosis when antemortem data are absent, reinforcing its essential role in unexpected death investigations.

This article was previously posted to Research Square (https://doi.org/10.21203/rs.3.rs-8229831/v1) on 15 December 2025, Preprint (Version 1).

## Case presentation

A 24-year-old male immigrant of Black ethnicity was referred to the Forensic Medicine department in Tripoli, Libya, in January 2024 as a case of unexpected death. The individual was pronounced dead upon arrival at the hospital. No previous clinical history or antemortem medical data were available.

The deceased's exterior examination revealed a slim physique, cachectic, standing 160 cm tall with a slumped posture. The head, neck, torso, and upper extremities showed no signs of trauma or fractures; however, the posterolateral region of the right thigh and upper lateral aspect of the left thigh had pressure ulcers or bedsores, findings consistent with prolonged immobilization or chronic illness.

**Figure 1:**
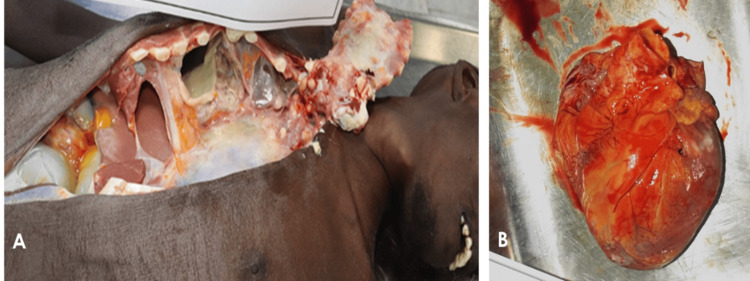
Autopsy: gross examination The deceased's exterior examination revealed a slim physique, cachexia, and both lungs and the pericardium showed purulent pathological alterations (A). The pericardium showed dense yellowish deposition on the external surface (B).

The autopsy examination revealed no anomalies in the scalp or skull, and the thoracic cavity contained no signs of bleeding or rib fractures. However, both lungs and the pericardium showed purulent pathological alterations, whereas the rest of the organs appeared normal (Figure 1A). Postmortem blood samples were collected for toxicology screening, while representative tissue biopsies from various organs were taken for histopathological examination.

**Figure 2:**
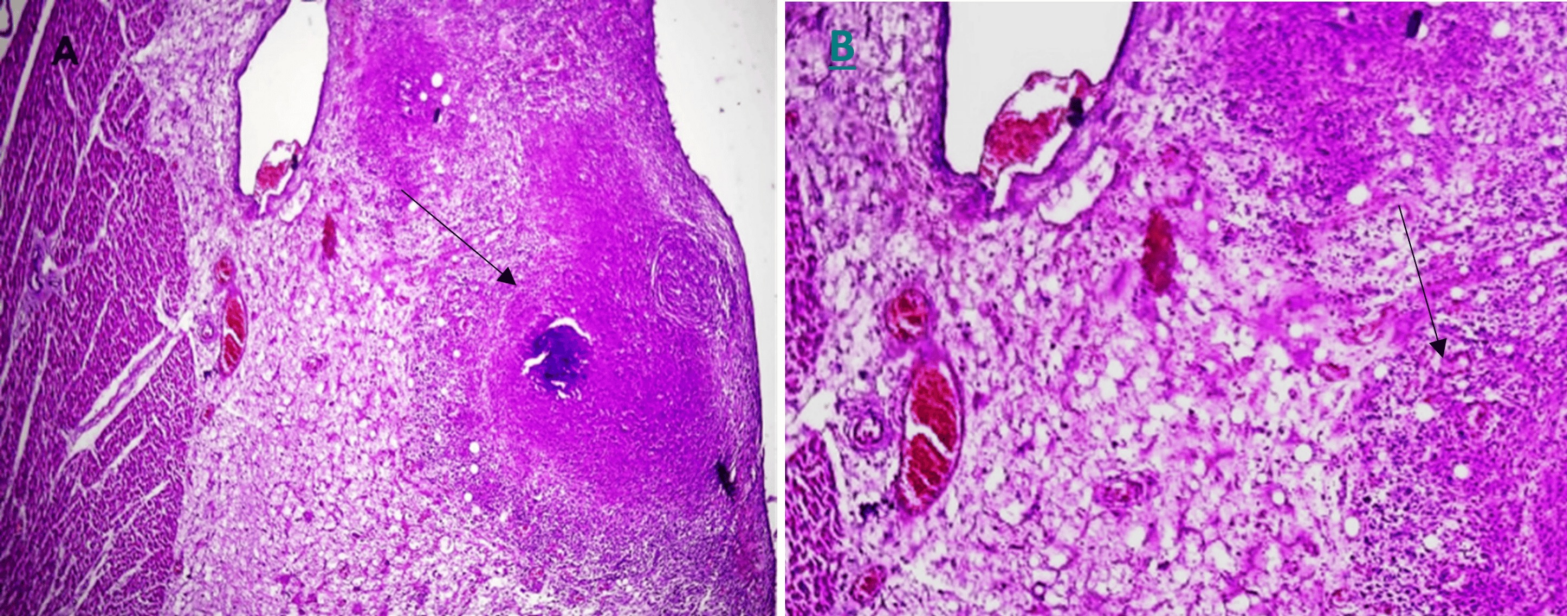
Histopathological examination of the pericardium showing tuberculous pericarditis (A) Chronic inflammation with central caseous necrosis in the pericardium, diagnostic for tuberculous pericarditis (H&E, ×20) (arrow). (B) Caseating granulomas with fibrin deposition and Langhans giant cells (arrow).

Histopathological evaluation of the submitted myocardium and coronary artery samples was found to be unremarkable. The pericardium, on the other hand, showed dense yellowish deposition on the external surface, thickened, opaque, with fibrinous exudate and possible adhesions (Figure 1B). Microscopically, granulomatous lesions with multinucleated giant cells, Langhans giant cells, lymphocytes, and epithelioid cells surrounding a core necrosis were visible, consistent with tuberculous pericarditis (Figure 2).

**Figure 3:**
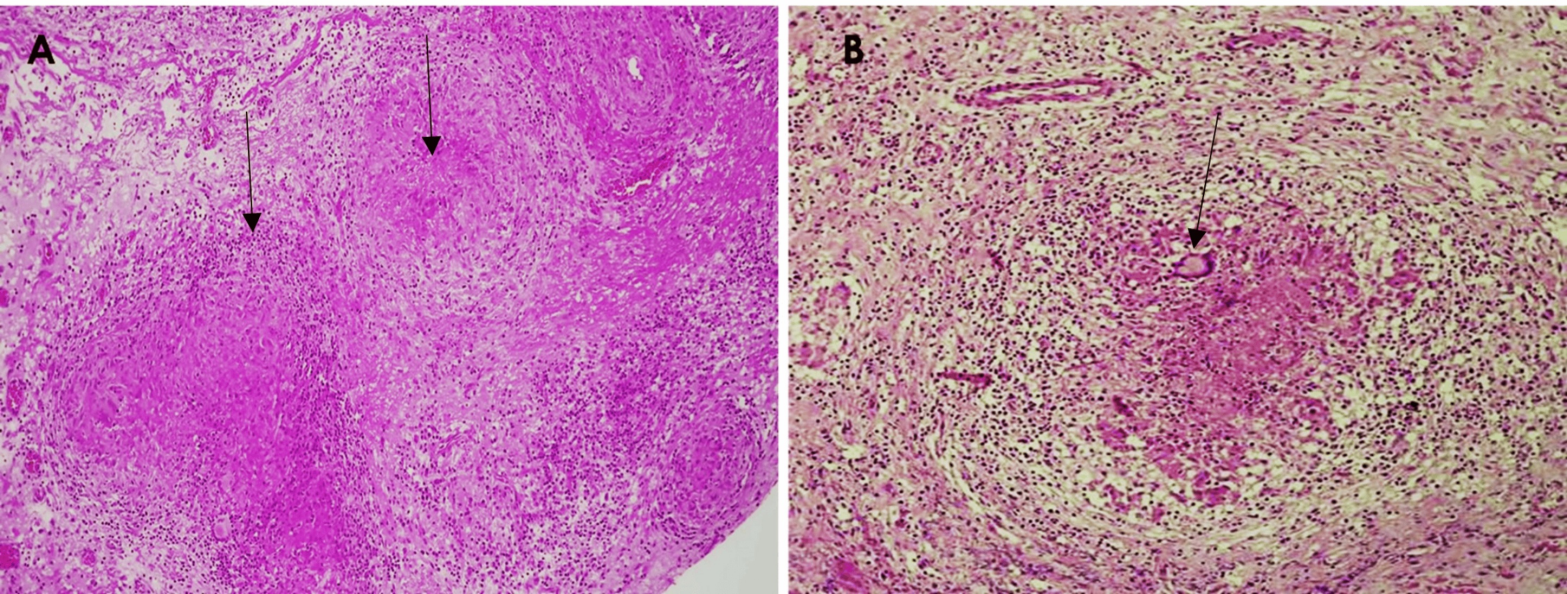
Histopathological examination of the lung showing pulmonary tuberculosis (A) Necrotizing granulomatous inflammation with central caseous necrosis (pink amorphous debris), characteristic of tuberculous infection (arrows) (H&E stain, ×20). (B) The granuloma is bordered by a fibrous cuff with chronic inflammatory infiltrates (i.e. epithelioid macrophages, lymphocytes, and Langhans giant cells (Arrow), characteristic of *Mycobacterium tuberculosis* infection.

Additionally, the lung tissue sections displayed extensive architectural damage and widespread granulomatous infiltrates (Figure 3), with multiple small granulomas distributed throughout the parenchyma--a pattern consistent with hematogenous dissemination. These findings support a diagnosis of disseminated tuberculosis.

Although acid-fast bacilli (AFB) staining represents the conclusive diagnostic test for tuberculosis, it could not be performed in this instance due to the unavailability of the requisite laboratory facilities. However, the morphological features are pathognomonic for tuberculosis, and other granulomatous diseases (such as sarcoidosis or fungal infections) were ruled out based on the presence of caseous necrosis. The Toxicology analysis revealed negative results for alcohol, drugs, and common poisons, thereby ruling out intoxication as a contributing cause of the death.

Postmortem examination with histopathological findings provided the definitive diagnosis and confirmed disseminated tuberculosis as the primary cause of death. The mechanical cause of death was likely a combination of respiratory failure from extensive pulmonary involvement and possible cardiac compromise from tuberculous pericarditis.

## Discussion

Tuberculous pericarditis is a particularly severe form of extrapulmonary tuberculosis, with mortality rates estimated between 17% and 40%, despite ongoing treatment efforts. The primary causes of death are often complications such as cardiac tamponade and constrictive pericarditis [10]. This case involves a 24-year-old male migrant who died unexpectedly from disseminated tuberculosis. Forensic histopathology was essential in establishing this diagnosis, confirming tuberculous pericarditis and extensive pulmonary tuberculosis as the causes of death. Without histopathological examination, this death would likely have been certified as “undetermined”. External causes, such as trauma or toxicological factors, were excluded. Histopathology revealed granulomatous inflammation with caseous necrosis in both the pericardium and lungs, characteristic of *M. tuberculosis* infection. The extensive pulmonary lesions represent the primary pathology leading to respiratory compromise and were the main driver of mortality. The lungs demonstrated widespread granulomatous involvement with multiple small coalescing lesions distributed throughout the parenchyma.

From a diagnostic standpoint, however, the pericardial results were just as important. The pericardial histology offered definitive evidence of tuberculous etiology in the absence of any antemortem clinical information. The granulomatous inflammation with central necrosis and multinucleated giant cells is pathognomonic for tuberculosis and allowed us to establish the cause of death with certainty. Additionally, systemic dissemination is confirmed by pericardial involvement, which is an essential finding for both medicolegal certification and public health surveillance. The concurrent lung and pericardium involvement, along with the histological findings, suggests disseminated tuberculosis, most likely hematogenous. Lymphatic or direct extension is less likely but cannot be ruled out. Bilateral, diffuse pulmonary granulomas support hematogenous dissemination, but pericardial involvement can occur through any of the three recognized routes.

The patient’s cachexia, pressure ulcers, and lack of accessible medical history suggest chronic illness and neglect, highlighting vulnerabilities commonly faced by underprivileged migrant groups. It is not unusual for vulnerable groups like refugees, criminals, and the socioeconomically disadvantaged to experience the deadly course of their disease, even in the absence of documented symptoms or medical assistance [11]. Delays or missed diagnoses are caused by several factors, including stigma around infectious diseases, underdiagnosis, a failure to seek medical attention, and restricted access to healthcare [12].

Socioeconomic status significantly affects disease diagnosis and outcomes. Low-income people are more vulnerable to tuberculosis due to overcrowded living conditions [13]. Studies indicate that men face a higher risk of unexpected or violent death as a result of advanced disease complications, possibly due to poor health-seeking behavior [13,14]. Many of these deaths occur outside healthcare facilities. For example, an autopsy research conducted in South Africa revealed a high prevalence of pulmonary TB in sudden at-home deaths, and was often linked to undetected HIV coinfection [15]. Similarly, 31% of forensic autopsy cases in Lusaka were found to be HIV-positive [14]. Given these reasons, targeted TB screening is essential for high-risk groups, including migrants, inhabitants of high-density/low-income areas, and HIV-positive individuals. This is especially relevant in Libya, which has become a major migratory hub in recent years. However, in our case, HIV co-infection could not be confirmed due to a lack of available lab testing, which is considered a limitation of this study.

The essential role of forensic histopathology in this case cannot be overstated. In the absence of any antemortem clinical data, toxicology results, or gross pathological findings specific enough to determine the cause of death, histopathological examination provided the definitive diagnosis. The granulomatous inflammation with caseous necrosis in both the pericardium and lungs is pathognomonic for tuberculosis [7]. This case serves as more evidence that forensic histopathology is an essential part of unexpected death investigations, especially in vulnerable communities where clinical history is frequently unavailable. The histological features observed in this case--granulomatous inflammation with caseous necrosis, multinucleated giant cells, Langhans giant cells, and epithelioid cell infiltrates--are hallmarks of tuberculosis and provide final diagnostic evidence. Although the autopsy indicated infection, histopathology conclusively verified it as the cause of death, highlighting its essential role in unexpected death investigations.

While diagnostic procedures, such as PCR and culture, are important, they can produce false negatives due to low bacillary load or prior antibiotic treatment [16]. Histopathology, on the other hand, reliably detects tuberculosis even in paucibacillary or extrapulmonary instances, such as tuberculous pericarditis and disseminated disease [16]. This indicates its crucial importance, especially in situations with limited resources where advanced molecular diagnostics are unavailable, and in forensic contexts where clinical history is absent. Also, other granulomatous conditions, including fungal infections and sarcoidosis, were taken into consideration. While fungal infections would show distinctive organisms on histology, sarcoidosis usually manifests as non-caseating granulomas. The etiology of tuberculosis is strongly supported by the occurrence of caseous necrosis. Histopathology continues to play an important role in lowering TB-related morbidity and death, particularly in high-risk and immunocompromised patients. In addition, the likelihood of HIV coinfection, while not investigated in this case, is clinically significant given Libya's increasing HIV prevalence and its reported link with disseminated and extrapulmonary TB presentations [17]. Future forensic investigations into similar instances should include multiplex disease screening to identify multifactorial contributors to mortality.

Diagnosis of TB in living patients usually involves clinical evaluation and lab/radiographic tests, though TB often goes undiagnosed until forensic autopsy. Postmortem TB detection is an important epidemiological indicator, and forensic autopsies are critical for TB mortality surveillance, exposing the burden of undiagnosed TB mortality. The prevalence of TB at forensic autopsies varies by region, as revealed in studies worldwide. Research in India reported a 5.1% TB rate at forensic autopsies, with 84.6% of cases undiagnosed before death [18]. In New Zealand, TB was shown to be the cause of death in 0.2% of autopsies, and a large proportion (70%) was undiagnosed before death [19]. Another study in Cape Town, South Africa, discovered a TB prevalence of 6.2% in cases of sudden unexpected death [20]. This variation may allude to regional differences in the prevalence of the infection. However, the burden of undiagnosed TB in community deaths has not been estimated in Libya yet.

It is important to distinguish between LTBI and active TB disease. LTBI is asymptomatic and non-infectious, with no risk of sudden death [7]. This case involves active, disseminated TB--a distinction that is critical for both clinical and forensic accuracy. This case also highlights the public health vulnerabilities in Libya due to the political instability and fragmented healthcare system contributing to TB underdiagnosis, especially among migrants and detainees. Undetected TB exacerbated by overcrowded settings where migrant populations are disproportionately affected, as demonstrated in this case. Addressing these vulnerabilities in high-risk settings like Libya presents a crucial opportunity to reduce unnecessary mortality.

## Conclusions

This case illustrates how forensic pathology, infectious disease trends, and health disparities intersect. It demonstrates that unexpected death in forensic practice may be the first indication of undiagnosed active disseminated tuberculosis, particularly in vulnerable migrant populations. Most importantly, this case demonstrates the crucial importance of forensic histopathology; without it, the cause of death would have been unknown. The combination of substantial lung pathology with diagnostically valuable pericardial findings indicates that histological examination is not optional, but rather essential in all unexpected mortality investigations. To avoid repeat incidents, public health initiatives must enhance early TB detection and healthcare access for vulnerable groups. Forensic institutions play a critical role by identifying and reporting such cases to guide public health interventions, including contact tracing to prevent further transmission.
